# The Potential Role of Cell-Death Mechanisms in the Pathogenesis of Familial Mediterranean Fever Attacks: Granzyme A and Beyond

**DOI:** 10.3390/diagnostics14182031

**Published:** 2024-09-13

**Authors:** Ece Yaglikara, Oguz Boluk, Yagmur Bayindir, Yelda Bilginer, Medine Aysin Tasar, Seza Ozen, Erdal Sag

**Affiliations:** 1 Department of Pediatrics, Ankara Training and Research Hospital, 06230 Ankara, Turkey; 2 Department of Pediatric Rheumatology, Hacettepe University, 06230 Ankara, Turkey; 3 Pediatric Rheumatology Unit, Translational Medicine Laboratories, Hacettepe University, 06230 Ankara, Turkey

**Keywords:** familial mediterranean fever, granzyme, pyroptosis

## Abstract

Background: FMF is the most common autoinflammatory disease. The activation of the pyrin inflammasome is the mainstay of the pathogenesis, which might lead to a specific cell-death mechanism, pyroptosis. Pyroptosis is a programmed inflammatory cell death mediated by gasdermin proteins, featuring cell swelling, membrane rupture, and release of inflammatory contents Aim: In this study we aimed to analyze the cell-death mechanisms in the pathogenesis of FMF attacks. Methods: Twenty-five FMF patients were included, and PFAPA patients (*n* = 10) and healthy controls (HC, *n* = 10) served as controls. We collected plasma samples from FMF and PFAPA patients during the attack and the attack-free period. We measured the soluble plasma levels of sFas, sFasL, granzyme A, granzyme B, perforin, granulysin, IL-2, IL-4, IL-10, IL-6, IL-17A, TNF-α, and IFN-γ by commercial pre-defined cytometric bead array kits. Results: There was no significant difference between groups in terms of sex and age between FMF patients and HCs, but PFAPA patients were younger than other groups due to the nature of the disease. We then analyzed the components of apoptosis and pyroptosis. The levels of sFasL (*p* = 0.035) and granzyme A (*p* = 0.038) in FMF patients were significantly increased during the attack period and decreased to levels comparable to HCs during the attack-free period. This increase was not seen in the PFAPA patients, with comparable levels with the HC group both during attack period and attack-free period. During the attack period of FMF patients, granzyme B (*p* = 0.145) and perforin (*p* = 0.203) levels were also increased; however, the differences were not statistically significant. The levels of sFasL, granzyme A, granzyme B, and perforin were closely correlated with each other during the attack period of FMF patients. Conclusions: Our study on death pathways during an FMF attack, suggests an upregulation in both pyroptosis through the granzyme-gasdermin pathway and apoptosis with the increased FasL and perforin levels, which was different from PFAPA patients. These findings might shed light on the reason for the nature of self-limited attacks, but further studies are needed to prove this hypothesis.

## 1. Introduction

Familial Mediterranean Fever is the most common autoinflammatory disease. It is most commonly seen in Armenians, Turks, and Jews [1]. The carrier frequency is very high with an expected disease prevalence of 1/1073 in Turkish people [2,3]. It is caused by autosomal recessive mutations in the *MEFV* gene encoding the pyrin protein [4,5]. Overactivation of the pyrin inflammasome leads to overproduction of IL-1b and subsequent proinflammatory cytokines, which in turn cause the clinical phenotype [6]. It is characterized by self-limited attacks of recurrent fever, abdominal pain, and arthritis lasting 1–3 days [7]. There are different criteria sets to classify patients with FMF. The first one was Tel–Hashomer criteria, which was proposed mainly for adult FMF patients [8]. Later, the Yalcinkaya–Ozen criteria was proposed for pediatric cases [9]. The most powerful aspect of the last set of criteria, known as the Eurofever/PRINTO criteria, is that it allows FMF classification to be made with only clinical parameters in centers where MEFV genetic analysis cannot be performed [10].

Pyrin is the essential protein in the inflammasome complex, causing excess inflammation via caspase-1 activation and IL-1 overproduction. According to Chae et al. [6], pyrin mutations in FMF knock-ins result in a gain-of-function via a caspase-1-dependent pathway and the apoptosis-associated speck-like protein containing CARD (ASC). Additionally, it has been demonstrated that pyrin promotes the activation of caspase-1 in response to pathogen alteration and the inactivation of RhoA-GTPases by various bacterial toxins [11]. Serine-threonine kinases (PKN1 and PKN2) triggered by RhoA bind and phosphorylate pyrin in healthy individuals. By binding to regulatory 14-3-3 proteins, phosphorylated pyrin inhibits the pyrin inflammasome. The majority of common and severe FMF mutations are clustered in the C terminal of the B30.2 domain, which is crucial for controlling pyrin phosphorylation because it prevents kinases like PKN1 from binding, and thus lowering the threshold for pyrin inflammasome activation [12]. The primary treatment for FMF remains colchicine, which improves quality of life, lowers the frequency of attacks, and prevents the consequence of secondary amyloidosis [13,14]. Colchicine both activates RhoA and reverses the inhibition of RhoA by bacterial toxins [12]. Since FMF is an inflammasomopathy and IL-1 plays a central role in the disease pathogenesis, anti-IL1 treatments are used in colchicine-resistant patients [15,16].

There are several studies regarding the activation of the pyrin inflammasome, but there is no data on why the attacks are self-limited and what ends these attacks. One of the hypotheses is the death of proinflammatory cells via a new mechanism of death called pyroptosis. The gasdermin family, specifically gasdermin D, causes pore formation on the cell surface, leading to the release of pro-inflammatory cytokines such as IL-1β and IL-18, and also cell swelling resulting in this inflammatory-induced cell death [17,18]. On the other hand, apoptosis is mediated by the interaction of the cell surface receptors Fas and FasL resulting in a non-inflammatory cell death. Since the attacks are self-limited and usually end in 2–3 days, pyroptosis, which induces inflammation together with apoptosis of active inflammatory cells, might be the reason behind the cessation of FMF attacks.

In this study, we aimed to assess pyroptosis and apoptosis in the pathogenesis of FMF attacks.

## 2. Material and Methods

Twenty-five FMF patients were included, and PFAPA patients (*n* = 10) and healthy controls (HC, *n* = 10) served as controls. All were from Ankara Training and Research Hospital, Department of Pediatric Rheumatology. We collected plasma samples from FMF and PFAPA patients during the attack and attack-free period. Fluids were immediately transferred to EDTA tubes, and plasma samples were stored at −80 °C. The acquisition, storage, and international sharing of samples had been approved by the Ankara Training and Research Hospital Ethics Committee for Non-Interventional Clinical Trials Ethics Committee with E-23-1208 number and informed written consent was obtained from the parents and patients. Clinical features of the patients (including demographics, Erythrocyte Sedimentation Rate (ESR), C-reactive protein (CRP), and hemogram) at the time of sample collection were also recorded.

We used a cytometric bead array to measure the soluble plasma levels of the cell-death markers. Cytometric bead arrays are bead-based immunoassays using the same basic principle as sandwich immunoassays. The size and intrinsic fluorescence intensity of beads allow for differentiation. Every set of beads functions as the capture beads for a certain analyte because it has an antibody attached to its surface. Each analyte will attach to its specific panel of capture beads when the panel is combined and treated with a sample containing target analytes specific to the capture antibodies. Following washing, a cocktail of biotinylated detection antibodies is added. Each detection antibody in the cocktail will attach to the unique analyte that is attached to the capture beads, creating sandwiches made of the capture bead, analyte, and detection antibody. The next step is to add streptavidin-phycoerythrin (SA-PE), which will bind to the biotinylated detection antibodies and provide fluorescent signals with intensities that correspond to the quantity of bound analytes. It is possible to separate analyte-specific populations and quantify the PE fluorescent signal, since the beads can be distinguished by size and internal fluorescence intensity on a flow cytometer. By employing a standard curve produced in the same test, the concentration of a certain analyte is determined. We measured the soluble plasma levels of sFas, sFasL, granzyme A, granzyme B, perforin, granulysin, IL-2, IL-4, IL-10, IL-6, IL-17A, TNF-α, and IFN-γ by the cytometric bead-based multiplex assay panel according to manufacturer’s instruction (LEGENDPLEX Human CD8/NK Panel catalogue number: 741065, Biolegend, San Diego, CA, USA) and analysed by a Novocyte 3005 flow cytometer.

### Statistical Analysis

Data were analyzed using GraphPad Statistics for MAC (Version 7.0). Descriptive statistics of baseline characteristics were summarized using median and interquartile range (IQR) for numeric variables and percentages for categorical variables. Differences in soluble levels of markers were tested by a Kruskal–Wallis analysis of variance and a Mann–Whitney U test. Post hoc tests were performed to explore significant differences between pairs and *p*-values were adjusted by using Bonferroni’s correction for multiple comparisons. Correlations between soluble levels were analyzed by Spearman’s rank correlation coefficient test. A *p*-value of 0.05 was considered statistically significant.

## 3. Results

There was no significant difference between groups in terms of sex (FMF, 52% male; PFAPA, 60% male; HC, 60% male; *p* = 0.664). The age of FMF patients and HC were comparable (FMF 8.5 ± 4.5 yrs vs. HC 9.9 ± 4.2 yrs 0.664), but PFAPA patients were younger (PFAPA 3.8 ± 2.1 yrs) than other groups due to the nature of the disease. The laboratory features of the patients during attack and attack-free period are given in Table 1.

**Figure 1:**
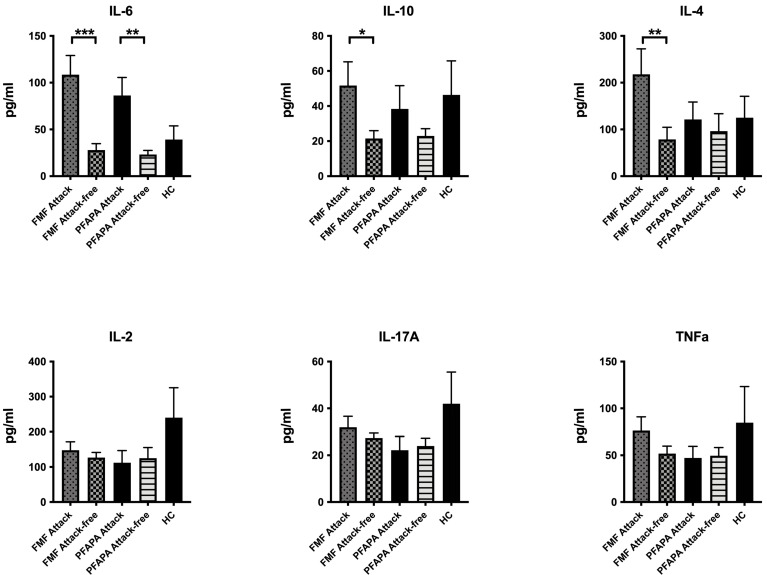
Plasma levels of different cytokines during attack and attack-free periods. FMF (familial mediterranean fever); PFAPA (periodic fever, aphthous stomatitis, pharyngitis, and adenitis); HC, (healthy control). (* *p* < 0.05; ** *p* < 0.01; *** *p* < 0.001).

When compared to attack-free period, IL-6 levels were higher during attacks of both FMF (*p* = 0.001) and PFAPA (*p* = 0.01) patients (Figure 1).

We then analyzed the components of apoptosis and pyroptosis. The levels of sFasL (attack, 93.4 (IQR 86.1) pg/mL vs. attack-free, 55.1 (IQR 28.1) pg/mL; *p* = 0.035) and granzyme A (attack, 2752.9 (IQR 3337.4) pg/mL vs. attack-free, 1354.7 (IQR 510.8) pg/mL; *p* = 0.038) were significantly increased during the attack period and became comparable to the levels of HCs during the attack-free period. This increase was not seen in the PFAPA patients, where they had comparable levels with the HC group both during attack period and attack-free period.

**Figure 2:**
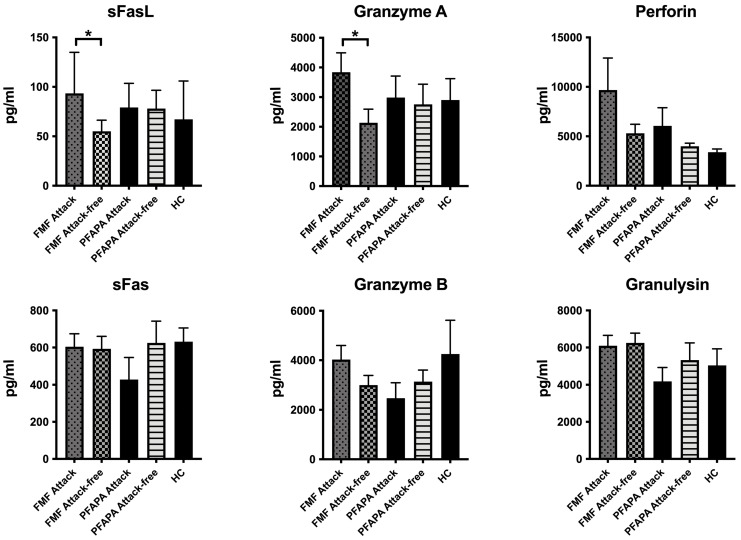
Plasma levels of apoptosis and pyroptosis markers during the attack and attack-free period. FMF (familial mediterranean fever); PFAPA (periodic fever, aphthous stomatitis, pharyngitis, and adenitis); HC, (healthy control). (* *p* < 0.05).

Granzyme B (attack, 3576.2 (IQR 2418) pg/mL vs. attack-free, 2304.6 (IQR 1946.7) pg/mL; *p* = 0.145) and perforin (attack, 4488.4 (IQR 6470.6) pg/mL vs. attack-free, 4605.9 (IQR 2998.8) pg/mL; *p* = 0.203) levels were also increased during the attack period of the FMF patients; however, these differences were not statistically significant (Figure 2).

**Figure 3:**
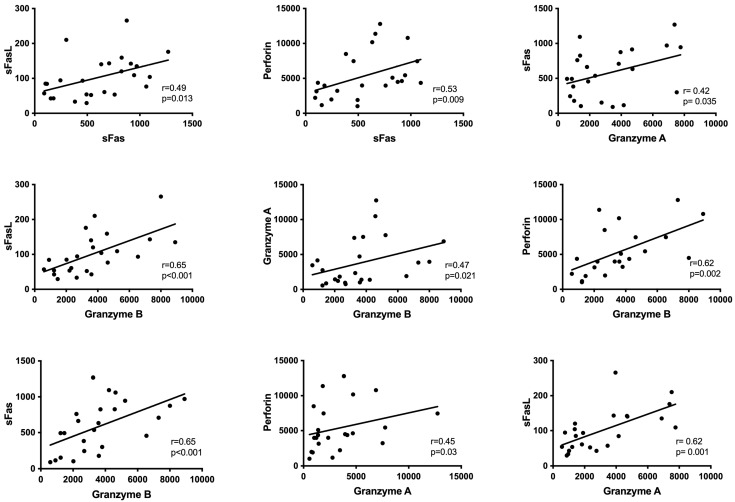
Correlations of the plasma levels of cell-death markers with each other.

The levels of sFasL, granzyme A, granzyme B, and perforin are closely correlated with each other during the attack period of FMF patients (Figure 3).

## 4. Discussion

Our study on death pathways during an FMF attack suggests an upregulation in both pyroptosis through the gasdermin pathway and apoptosis with the increased FasL and perforin levels.

FMF is the most common autoinflammatory disease, characterized by clinical attacks of fever and serosal inflammation that last for 1–3 days [7]. Severe abdominal pain, chest pain and/or arthritis reflect serosal inflammation. In the attack-free period, the elevated acute phase reactants reflect the ongoing subclinical inflammation in untreated patients. Diagnosis is confirmed by mutation analysis of the Mediterranean Fever (MEFV) gene.

FMF is caused by gain-of-function mutations in the MEFV gene encoding the pyrin protein [7]. Pyrin is an element of the inflammasome. Increased activation of the pyrin inflammasome remains at the center of the complex pathogenesis [12]. Work from Dr Shao’s lab has shown that in response to pathogen alteration and RhoA-GTPase inactivation by several bacterial toxins, pyrin promotes caspase-1 activation [11]. Serine-threonine kinases (PKN1 and PKN2) triggered by RhoA bind and phosphorylate pyrin in healthy individuals. By binding to regulatory 14-3-3 proteins, phosphorylated pyrin inhibits the pyrin inflammasome. The majority of common and severe FMF mutations are clustered in the C terminal of the B30.2 domain, which is crucial for controlling pyrin phosphorylation because it prevents kinases like PKN1 from binding, lowering the threshold for pyrin inflammasome activation [12]. The activation of the pyrin inflammasome hence leads to the cleavage of procaspase and subsequently caspase-1 activation and IL-1b production. The IL1 produced is secreted through the pores mediated by pyroptosis and gasdermins, which play an important role in this mechanism [17,19]. Our results highlight the importance of the gasdermin pathway during inflammatory attacks. On the other hand, it is tempting to speculate that the activation of apoptosis may define the short duration of attacks in FMF. There are some recent studies aiming to define more in-depth pathways that might explain different effectors for the self-limited attacks. The lifespan of neutrophils is shorter than one day but might be long with a maximum of five days, and the attacks of FMF usually subside in 2–3 days [20,21]. Thus, an exaggerated apoptosis may be contributing to the limitation of the attack duration. This increase was not evident in the PFAPA patients, where the attack usually lasts for 4–5 days [22,23].

The terms “pyro” and “ptosis”, which stand for the characteristics of inflammation (fire or fever) and planned cell death (falling), respectively, were originally used to propose the name “pyroptosis” in 2001 [24]. Pyroptosis has been redefined as a type of programmed cell death mediated by gasdermin proteins with the features of cell swelling, membrane rupture, and the release of cellular contents as a result of the identification of several pyroptosis-executing proteins [19,25,26,27]. Pyroptosis is an inflammatory cell death mechanism, using two pathways. The canonical pathway responds to pathogen-associated molecular patterns (PAMPs) and damage associated molecular patterns (DAMPs) while the non-canonical pathway responds to intracellular LPS from Gram-negative bacteria. Gasdermin D (GSDMD) is cut into two pieces by caspase-1 in the canonical pathway and by caspase-4/5 in noncanonical pathways, which forms pores in the membranes of cells leading to the release of pro-inflammatory cytokines such as IL-1β and IL-18, disturbing ionic balance, followed by water influx, ultimately resulting in cell swelling, culminating into inflammatory induced cell death [17,18].

In studies with FMF mouse models (Mefv^V726A/V726A^), ex vivo-infected macrophages exhibited synchronized GSDMD cleavage in conjunction with IL-β with *C. difficile* infection, indicating that cytokine release may result in IL-1 beta-dependent autoinflammation. Moreover, deletion of GSDMD in vivo completely rescued growth retardation, anemia, cytokine production, neutrophilia, and tissue damage secondary to autoinflammation seen in MefvV726A/V726A mice [28].

The gasdermin-D family, which operates through caspase activation, has been previously shown to be active in FMF patients. Our study specifically detected increased granzyme A levels in FMF patients during attack periods. Granzyme A is a gasdermin-B family molecule that induces pyroptosis without activating the caspase pathway; it is mostly generated by cytotoxic T lymphocytes and natural killer (NK) cells [29]. Simultaneously, statistically significant increases in FasL levels were noted during the FMF attacks, which subsequently returned to baseline levels. The regulation of cell life and death is largely dependent on the Fas/FasL system. On the other hand, no appreciable variations were observed in the levels of granzyme A and FasL in the PFAPA patients before and after attacks. Additionally, our research showed that during attacks in FMF patients, not only did granzyme A and sFasL levels rise, but also granzyme B and perforin levels also, albeit not to a statistically significant degree. The fact that these compounds exhibited statistically significant correlations with one another is noteworthy because it suggests that NK and cytotoxic T cells are involved in the pathophysiology of FMF attacks.

Uncontrolled caspase 1 activation and IL-1β release have been identified as the most important processes in FMF. Due to the NLRP3 mutation, this pattern is comparable to that seen in Cryopyrin-Associated Periodic Syndromes (CAPS), which suggests that patients with FMF may have distinct autoinflammatory pathways. In this regard, cells from patients with FMF had high levels of S100A8 and S100A9 alarmins but not cells from patients with other inflammatory disorders like sepsis, where these molecules act as ligands for TLR4 and cause strong inflammatory responses within tissues. Furthermore, the pyrin/caspase1/GSDMD dependent pathway serves as a distinct pathway for the release of alarmins produced from S100, underscoring their importance in the initiation and sustenance of autoinflammation in FMF pathogenesis [30].

While pyroptosis is an inflammatory cell death, apoptosis is non-inflammatory. The increased apoptotic signals during FMF attacks may suggest their role in limiting the clinical attacks since neutrophils are required in the sites of inflammation. Increased apoptosis in FMF has been suggested before in a study by Ozen et al. [31] where the authors reported that neutrophil and monocyte apoptosis was significantly increased during the attack by analyzing Fas and FasL cellular expression [31]. These results also support our findings. Further studies are needed to confirm our results, which may offer an explanation of the clinical attacks on the surface. However, subclinical inflammation will continue in untreated patients.

In terms of cytokines, serum IL-6 levels were elevated both in FMF patients and PFAPA patients during attack period, which was not a surprise since IL-6 is one of the main pro-inflammatory cytokines. Moreover, we have seen that IL-17 and TNFa levels were also elevated during FMF attacks, but these increases were not statistically significant.

Our study has some limitations as well. We studied only the plasma levels of cell-death markers but did not perform cell surface expression of these markers and functional studies. We included a relatively limited number of patients, and we could not study caspases together with other cell-death markers.

## 5. Conclusions

Our study reveals the importance of apoptosis and pyroptosis, specifically granzyme A, which is a caspase-independent activator of pyroptosis, in the mechanism of FMF attacks, which was different from PFAPA patients. These findings might shed light on the reason for the nature of self-limited attacks, but further studies are needed to prove this hypothesis.

## Figures and Tables

**Table 1:** Laboratory parameters of patients during attack and attack-free period.

	FMF Attack	FMF Attack-Free	*p*	PFAPA Attack	PFAPA Attack-Free	*p*
Hemoglobin (g/dL)	12.6 ± 1.0	12.71 ± 1.30	0.705	10.7 ± 3.6	11.9 ± 0.9	0.966
WBC (/mm^3^)	10,662.4 ± 4937.3	8187.90 ± 4625.12	0.074	11,376.0 ± 4128.7	10,237.0 ± 3957.6	0.753
Platelet (10^3^/mm^3^)	287,040 ± 102,403	288,480 ± 75,212	0.955	309,200 ± 144,024	384,600 ± 74,099	0.159
Erythrocyte sedimentation rate (mm/hr)	24.3 ± 15.1	10.45 ± 6.12	<0.001	13.9 ± 5.8	11.9 ± 10.8	0.631
C-reactive protein(mg/dL)	68.8 ± 69.0	6.00 ± 9.73	<0.001	63.4 ± 56.9	12.0 ± 17.9	0.021

## Data Availability

The data presented in this study are available on request from the corresponding author. The data are not publicly available due to confidentiality issues.
